# High‐density genomic data reveal fine‐scale population structure and pronounced islands of adaptive divergence in lake whitefish (*Coregonus clupeaformis*) from Lake Michigan

**DOI:** 10.1111/eva.13475

**Published:** 2022-09-20

**Authors:** Yue Shi, Jared J. Homola, Peter T. Euclide, Daniel A. Isermann, David C. Caroffino, Megan V. McPhee, Wesley A. Larson

**Affiliations:** ^1^ College of Fisheries and Ocean Sciences University of Alaska Fairbanks Juneau Alaska USA; ^2^ Wisconsin Cooperative Fishery Research Unit, College of Natural Resources University of Wisconsin‐Stevens Point Stevens Point Wisconsin USA; ^3^ U.S. Geological Survey, Wisconsin Cooperative Fishery Research Unit, College of Natural Resources University of Wisconsin‐Stevens Point Stevens Point Wisconsin USA; ^4^ Michigan Department of Natural Resources Charlevoix Research Station Charlevoix Michigan USA; ^5^ National Marine Fisheries Service, Alaska Fisheries Science Center, Auke Bay Laboratories National Oceanic and Atmospheric Administration Juneau Alaska USA

**Keywords:** adaptive divergence, genome scan, Lake Michigan, lake whitefish, population structure, rapture

## Abstract

Understanding patterns of genetic structure and adaptive variation in natural populations is crucial for informing conservation and management. Past genetic research using 11 microsatellite loci identified six genetic stocks of lake whitefish (*Coregonus clupeaformis*) within Lake Michigan, USA. However, ambiguity in genetic stock assignments suggested those neutral microsatellite markers did not provide adequate power for delineating lake whitefish stocks in this system, prompting calls for a genomics approach to investigate stock structure. Here, we generated a dense genomic dataset to characterize population structure and investigate patterns of neutral and adaptive genetic diversity among lake whitefish populations in Lake Michigan. Using Rapture sequencing, we genotyped 829 individuals collected from 17 baseline populations at 197,588 SNP markers after quality filtering. Although the overall pattern of genetic structure was similar to the previous microsatellite study, our genomic data provided several novel insights. Our results indicated a large genetic break between the northwestern and eastern sides of Lake Michigan, and we found a much greater level of population structure on the eastern side compared to the northwestern side. Collectively, we observed five genomic islands of adaptive divergence on five different chromosomes. Each island displayed a different pattern of population structure, suggesting that combinations of genotypes at these adaptive regions are facilitating local adaptation to spatially heterogenous selection pressures. Additionally, we identified a large linkage disequilibrium block of ~8.5 Mb on chromosome 20 that is suggestive of a putative inversion but with a low frequency of the minor haplotype. Our study provides a comprehensive assessment of population structure and adaptive variation that can help inform the management of Lake Michigan's lake whitefish fishery and highlights the utility of incorporating adaptive loci into fisheries management.

## 1 INTRODUCTION

Understanding patterns of genetic structure in natural populations and using this information to define genetic management units is an integral component of the effective management of within‐species diversity (Moritz, 1994). Historically, delineation of genetic management units has primarily relied on genotypes from relatively few (10s to 100s) neutral markers, but this has changed drastically in the last decade with the proliferation of genomic datasets containing information from thousands to millions of genetic markers (Allendorf et al., 2010). These genomic resources facilitate the identification of putatively adaptive markers that have great potential for improving the resolution of conservation units and enhancing population assignment success (Funk et al., 2012; Nielsen et al., 2012). While there are debates surrounding conservation measures focused on preserving diversity at single genes (Kardos et al., 2021; Kardos & Shafer, 2018; Waples & Lindley, 2018), mounting evidence suggests that understanding the landscape of adaptive diversity is of great value for developing effective conservation strategies and genetic monitoring tools that promote resiliency in a rapidly changing world (Bay et al., 2018; Razgour et al., 2019; Schwartz et al., 2007).

Examination of genome‐wide variation among populations indicates that genomic differentiation can be relatively uniform across the genome or localized in specific genomic regions, commonly referred to as genomic islands of differentiation (Johannesson et al., 2020; Nosil et al., 2009; Roda et al., 2017; Strasburg et al., 2012; Thompson et al., 2020). Although many factors such as genetic incompatibilities, variation in recombination, mutation rate, and gene density can lead to a heterogeneous genomic landscape of differentiation (Ravinet et al., 2017; Schumer et al., 2018), divergent selection and adaptation are often implicated because as populations diverge, genomic regions associated with adaptation should theoretically display elevated differentiation (Via & West, 2008). Therefore, evidence of nonrandom distribution of outlier loci across the genome increases confidence in identifying signals of natural selection. Because of this, it is important to genotype a large number of markers (generally hundreds of thousands to millions) to ensure that significant regions of adaptive divergence are accurately identified (Benjelloun et al., 2019; Lowry et al., 2017).

Lake whitefish (*Coregonus clupeaformis*) in the Laurentian Great Lakes of North America are an excellent model for investigating adaptive differentiation as they spawn in heterogenous habitats, ranging from eutrophic tributaries to oligotrophic rocky reefs, and at least some lake whitefish exhibit spawning site fidelity (Ebener et al., 2010). Differences in spawning site‐specific selection pressures may contribute to variations in metrics such as length, age distribution, weight at length, fecundity and growth among spawning populations (Isermann et al., 2020). Lake whitefish also represent an economically and culturally important resource throughout the region (Ebener et al., 2008). The Lake Michigan lake whitefish fishery's yield declined to all‐time lows in all Great Lakes in the 1960s due to a combination of factors including overfishing, habitat degradation, and invasion of sea lamprey *Petromyzon marinus* (Ebener et al., 2008; Jensen, 1976). In response, management agencies took a series of actions to regulate commercial fisheries, restore habitat, and control invasive species (Ebener et al., 2008). The resulting recovery of this species in the early 1990s was considered a substantial management success. However, starting in the mid‐1990s, multiple spawning stocks of lake whitefish in various Great Lakes experienced recruitment failures due to uncertain causes, whereas stocks from Green Bay (northwestern Lake Michigan) appear to be flourishing (Ebener et al., 2021). Because genetic changes brought by fisheries exploitation are not obvious and occur at a modest rate (Law, 2007), ensuring long‐term productivity of a species depends upon conserving all subpopulations via portfolio effects (Schindler et al., 2010). Additionally, stock‐specific management strategies can be difficult to effectively implement if a species is harvested in mixed‐stock fisheries stocks. Therefore, the identification of genetic management units and management strategies that ensure the viability of each unit is vital for the long‐term sustainability of lake whitefish fisheries.

Past genetic research using 11 microsatellite loci identified six genetic stocks of lake whitefish within Lake Michigan (VanDeHey et al., 2009), with two stocks on the northwestern side and four stocks on the eastern side. However, low pairwise *F*_ST_ values (VanDeHey et al., 2009) and ambiguity in genetic stock assignments (Isermann et al., 2020) suggest a limited number of neutral microsatellite markers do not provide adequate power for delineating Lake Michigan lake whitefish stocks, prompting calls for genomics approaches to investigate stock structure. Moreover, genomic approaches may be useful for identifying locally adaptive variants that are key to long‐term species persistence but that could be overlooked when establishing management units based on neutral or genome‐wide variation (Funk et al., 2012; Nielsen et al., 2012). In species that experience high gene flow across heterogeneous environments, likely the case for lake whitefish in Lake Michigan, adaptive loci are expected to be tightly clustered, generating pronounced genomic islands of adaptive divergence (Shi et al., 2021).

Here, we generated a dense genomic dataset for lake whitefish from Lake Michigan, USA, to investigate their spatial genetic structure and genomic patterns of adaptive differentiation. Signals of adaptive divergence were assessed using the full dataset and geographic region‐specific datasets. Our work provides a baseline understanding of the genetic structure of lake whitefish populations in Lake Michigan and establishes a foundation for future research and management actions that could help improve the conservation outlook for this regionally important native species.

## 2 METHODS

### 2.1 Rapture panel development

Rapture, also known as RAD capture, is a sequence capture approach and can generate a more reproducible set of genetic markers at a much lower sequencing cost per sample compared to regular restriction site‐associated DNA (RAD) sequencing (Ali et al., 2016; Meek & Larson, 2019). We developed a Rapture bait panel for lake whitefish by conducting preliminary RAD sequencing on 30 samples representing six individuals from each of the five Great Lakes (Tables S1 and S5). Therefore, this Rapture panel should avoid issues associated with ascertainment bias (Lachance & Tishkoff, 2013) for applications in any of the Great Lakes.

DNA was isolated from fin clip samples preserved in >95% ethanol using Qiagen DNeasy Blood and Tissue Kits. DNA extracts were quantified and normalized to 20 ng/μl. A single RAD library was prepared using the restriction enzyme *PstI* and NEBNext® Ultra™ DNA Library Prep Kit for Illumina® following the BestRAD protocol (Ali et al., 2016) as detailed in Ackiss et al. (2020). The prepared RAD library was sent to Novogene (Sacramento, CA) for sequencing on the Illumina HiSeq4000 platform (PE 150). Raw RAD sequences were processed using STACKS v2.3 (Rochette et al., 2019) following the protocol detailed in Ackiss et al. (2020) (‐m 3, ‐M 5, ‐n 3). SNP filtering was performed with VCFtools v0.1.16 (Danecek et al., 2011) and included (1) removing loci with a minor allele count less than 3 and (2) genotyped in fewer than 30% of individuals, and (3) removing individuals missing more than 90% of loci. We kept only 1 SNP per RAD tag using the *thin* command in VCFtools (‐‐thin 400). We then used HDPlot (McKinney et al., 2016) to remove any putatively paralogous loci, which were loci with heterozygosity greater than 0.55 or a read ratio deviation greater than 5 or less than −5. All loci that passed filtering were aligned to the reference genome of a closely related species, the European Alpine whitefish (*Coregonus* sp. ‘*balchen*’; GCA_902810595.1). We removed loci with mapping quality less than 30 and loci that were mapped to multiple alignment positions. Additionally, only one locus every ~20 Kb was retained. Bait development (80 nt baits with 2× tiling density) was conducted by Arbor Biosciences (Ann Arbor, MI) and only the baits closest to the 5′ ends for each locus were kept.

### 2.2 Rapture sequencing, SNP discovery, and genotyping

**FIGURE 1:**
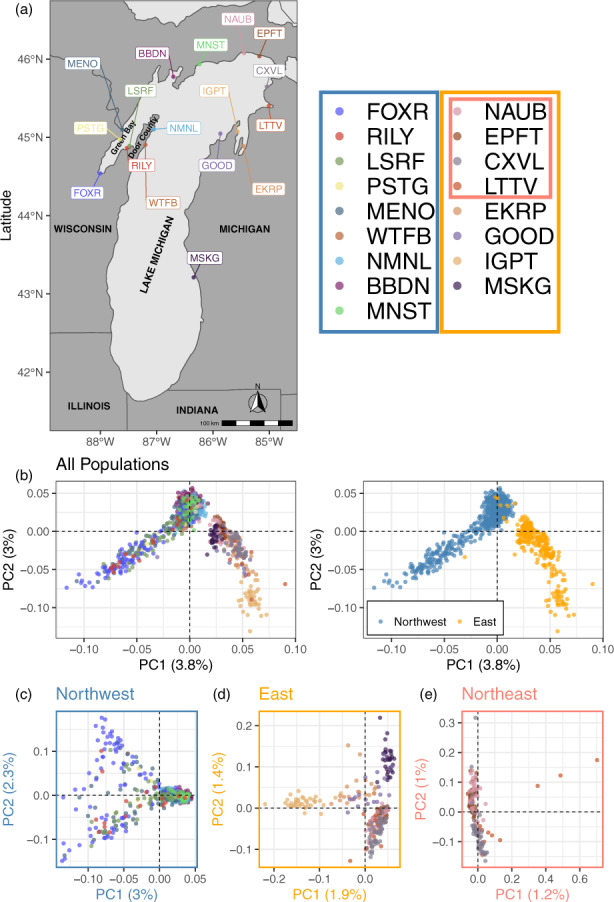
Population structure analysis of the 17 populations of lake whitefish (*Coregonus clupeaformis*) sampled across Lake Michigan. Details about the sampling sites are provided in Table 1. (a) Shows the geographic locations of the 17 populations in Lake Michigan. Populations in the legend were boxed and color coded to represent their geographical subset. Individual‐based principal component analyses (PCA) using all 197,588 SNPs shows the overall population structure with the largest genetic break between northwestern and eastern sides of Lake Michigan (b). Regional population structure in the northwestern (c), eastern (d) and northeastern Lake Michigan (e) were also shown.

Using the rapture panel that we developed, we sequenced 951 baseline samples collected during the spawning seasons from 2014–2019 from 19 known spawning locations in Lake Michigan (Figure 1a, Table 1). Samples from Arcadia and Ludington were discarded due to small sample sizes (≤2 individuals). Among the remaining 17 baseline populations, there was an average of 56 individuals per population and the sample size ranged from 10–130 individuals (Table 1).

**TABLE 1:** Summary of sampled Lake Michigan lake whitefish population locations and estimates of their genetic diversity based on 197,588 single nucleotide polymorphisms across 92,725 restriction site associated DNA sequencing loci

Population	Code	Lake Region	Latitude	Longitude	Sampling Year	*N*_collected_	*N*_genotyped_	*H*_E_	*H*_O_	*F*_IS_
Arcadia	ARCA		44.49754	−86.2649	2018	2	0			
Big Bay de Noc	BBDN	Northwest	45.77378	−86.7038	2017, 2018	115	90	0.124	0.146	−0.049
Cross Village	CXVL	Northeast and East	45.64815	−85.0439	2019	50	49	0.122	0.144	−0.048
Elk Rapids	EKRP	East	44.89042	−85.4592	2018	34	23	0.117	0.144	−0.054
Epoufette	EPFT	Northeast and East	46.03675	−85.1758	2018	48	48	0.119	0.146	−0.06
Fox River	FOXR	Northwest	44.54018	−88.0037	2017	98	94	0.127	0.148	−0.046
Good Harbor Bay	GOOD	East	45.0486	‐85.8689	2018	23	19	0.118	0.146	−0.055
Ingalls Point	IGPT	East	45.07346	−85.5644	2019	48	38	0.129	0.161	−0.072
Larson's Reef	LSRF	Northwest	44.89017	−87.4824	2019	48	43	0.134	0.177	−0.101
Little Traverse Bay	LTTV	Northeast and East	45.40268	−85.0091	2019	14	11	0.122	0.163	−0.074
Ludington	LUDI		43.95548	−86.4893	2018	1	0			
Manistique	MNST	Northwest	45.93338	−86.2383	2019	48	42	0.127	0.16	−0.076
Menominee River	MENO	Northwest	45.09851	−87.6107	2015, 2018	130	122	0.125	0.148	−0.051
Muskegon	MSKG	East	43.21106	−86.3435	2018	50	33	0.119	0.154	−0.076
Naubinway	MAUB	Northeast and East	46.07598	−85.4492	2019	40	37	0.123	0.153	−0.067
North/Moonlight Bays	NMNL	Northwest	45.10253	−87.049	2014, 2017	125	104	0.123	0.15	−0.063
Peshtigo River	PSTG	Northwest	44.97299	−87.654	2017	10	9	0.127	0.167	−0.067
Rileys Bay	RILY	Northwest	44.86496	−87.5275	2019	17	17	0.13	0.17	−0.081
Whitefish Bay	WTFB	Northwest	44.90512	−87.1999	2015	50	50	0.125	0.146	−0.046

*Note*: Populations from Arcadia and Ludington were excluded from analyses following sequence quality filtering.

Abbreviations: Code, acronyms for each population; *N*_collected_, number of individuals collected; *N*_genotyped_, number of individuals genotyped; *H*_*E*_, expected heterzygosity; *H*_*O*_, observed heterzygosity; *F*_*IS*_, inbreeding coefficient.

DNA was isolated from fin clip samples preserved in >95% ethanol with Qiagen DNeasy Blood and Tissue Kits. RADseq libraries were prepared in the same way as the initial RAD sequencing for the Rapture panel development, and bait capture was conducted following the myBaits protocol v.4.01 (https://arborbiosci.com/mybaits‐manual/) and Euclide et al. (2021) with an annealing temperature of 60 °C for library amplification. A total of 12 Rapture libraries were sent to Novogene (Sacramento, CA) for sequencing on two Illumina NovaSeq S4 lanes (PE 150).

Reads were demultiplexed and trimmed using the *process_radtags* module in STACKS (parameter flags: ‐c ‐q ‐r ‐‐filter_illumina ‐‐bestrad ‐t 140). Adaptor sequences were clipped from reads using *trimmomatic* v0.39 (Bolger et al., 2014) with the following parameters: ILLUMINACLIP:2:30:10 SLIDINGWINDOW:4:15 MINLEN:50 and the adaptor sequences fasta file provided with *trimmomatic*, TruSeq‐3‐PE‐2.fa. Demultiplexed and trimmed reads were mapped to the European Alpine whitefish reference genome (De‐Kayne et al., 2020) using bwa‐mem v0.7.17 with default settings (Li, 2013). Mapped BAM files were sorted and filtered using SAMtools view v1.10 (Li et al., 2009) to exclude reads with mapping quality <20 and reads not mapped in proper pairs (parameters flags: ‐q 20 ‐f 2 ‐F 2308).

Mapped reads were genotyped and PCR duplicates were removed (−‐rm‐pcr‐dups) using the *gstacks* module in STACKS. The *populations* module was used to produce a VCF file that included SNPs and haplotypes genotyped in greater than 30% of individuals (parameter flags: ‐R 0.3 ‐H). Called SNPs were filtered with VCFtools to remove SNPs with a minor allele count of less than 3 and SNPs genotyped in fewer than 70% of individuals. Individuals missing more than 50% of retained SNPs were also removed. Putative sample duplicates were identified by calculating relatedness between samples using VCFtools (‐‐relatedness). We estimated relatedness only using loci with minor allele frequency (MAF) ≥ 0.05 to avoid upward bias and we did not identify any sample duplicates with a relatedness greater than 0.9. We further removed RAD loci with more than 10 haplotypes (Bootsma et al., 2020) using VCFtools. A final SNP dataset was generated by rerunning the *populations* module with the retained RAD loci.

### 2.3 Population structure and genetic diversity

To assess the extent of genetic structure among populations, we performed an individual‐based principal component analysis (PCA) using PLINK v2.0 (Purcell et al., 2007). We then used hierarchical PCAs to further investigate regional patterns of population structure in northwestern, eastern and northeastern Lake Michigan separately. In addition, we estimated the number of ancestral populations, K, contributing to the observed genetic clustering using the program ADMIXTURE v1.3.0 (Alexander & Lange, 2011). We tested K from 1 to 10 with ADMIXTURE's cross‐validation procedure (‐‐cv = 10) to examine the support for each K value. Similar to PCA, we conducted ADMIXTURE analyses in northwestern and eastern Lake Michigan separately.

To estimate genetic differentiation among populations, we calculated pairwise *F*_ST_ (Weir & Cockerham, 1984) in Arlequin v3.5.2 and tested for significance using 10,000 permutations per test (Bonferroni‐corrected *α* = 0.05/136 with a total of 136 pairwise comparisons). Overall weighted *F*_ST_ (Weir & Cockerham, 1984) was calculated using PLINK (‐‐fst ‐‐family). We also tested for isolation by distance or IBD (Wright, 1943) by conducting a linear regression of linearized pairwise *F*_ST_, or *F*_ST_/(1−*F*_ST_), to the in‐water distance between populations. The in‐water distances corresponded to the length of the shortest, direct within‐water path between two given locations, and were computed as least‐cost path distances between all pairs of populations by assigning infinite resistance to land areas and resistance of one to water using the function *costDistance* of the R package *gdistance* v1.1.1 (van Etten, 2017). The statistical significance of IBD was evaluated with a Mantel test (Pearson's product moment correlation; 10,000 permutations) using the R package *vegan* v2.5.6 (Oksanen et al., 2020). We tested for IBD on the full dataset and also based only on pairwise comparisons of populations belonging to either the northwestern or eastern side of the lake. Since the southernmost Muskegon (MSKG) is a geographic outlier, we also tested for IBD after removing MSKG.

To assess genetic diversity, we estimated the mean expected (*H*_E_) and observed (*H*_O_) heterozygosity, and the mean measure of inbreeding coefficient (*F*_IS_) for each population using the population.sumstats_summary.tsv file produced by the *populations* module in STACKS. Effective population size (*N*_e_) was estimated in each population using the program NeEstimator v2.1(Do et al., 2014) with a *p*_crit_ of 0.05. We used the “LD locus pairing” option to correct for the effects of physical linkage on the estimates of *N*_e_ by restricting pairings to loci on different chromosomes (Waples et al., 2016). To reduce the computational demand for *N*_e_ calculation, we thinned the dataset by retaining SNPs with MAF ≥ 0.05 in the whole dataset, located at least 400 bp from one another, and genotyped in every population. To further reduce the size of the dataset so that it could be analyzed by the program, we randomly selected 10,000 SNPs from the remaining SNPs.

### 2.4 Identification of Genomic Islands of adaptive divergence

We performed a genome scan to identify genomic islands of adaptive divergence using *pcadapt* v4.3.1 (Luu et al., 2016) and following the best practices (neutral parameterization with thinned SNPs and genome scan on all SNPs) described in Lotterhos (2019). SNP thinning was conducted using the function *snp_ autoSVD* (min.mac = 3, max.iter = 10, roll.size = 0) in the R packages *bigsnpr* v1.8.7 (Prive et al., 2018), which uses sliding windows to remove SNPs with correlation coefficients >0.2 with the SNP of the highest MAF in each window and remove regions with putative long‐range LD. The thinned set of SNPs was used to determine a K value based on a visual examination of a scree plot. The determined K value (Table S2) was then used on all SNPs to compute *p* values to test for outliers (min.maf = 0.05). We adjusted the resulting *p* values for false discovery rate by computing *q* values using the R package *qvalue* v2.16.0 (Lai, 2017). An SNP was considered as an outlier if *q* < 0.01. Since the genetic signal of fine‐scale adaptive differentiation could be masked by the overall population differentiation, we also conducted *pcadapt* analysis on populations in each geographical region separately (i.e. northwestern, eastern and northeastern Lake Michigan) following the same procedure as described above. The outliers identified by *pcadapt* in either the full dataset or the region‐specific dataset were termed “*pcadapt* outliers”.

To identify genomic regions enriched for *pcadapt* outliers, likely to be the candidate regions for genomic islands of adaptive divergence, we conducted a sliding window analysis across the genome using a window size of 250 SNPs and a step size of 50 SNPs. Chromosome 40 was removed from the sliding window analysis as it was very small and only contained 73 SNPs. We defined windows with at least 10 *pcadapt* outliers (*q* < 0.01) as candidate windows under selection. Consecutive candidate windows were combined into candidate regions. To reduce the effect of window size on defining the size of candidate regions, we used the minimum *pcadapt* outlier SNP position as the start position and the maximum *pcadapt* outlier SNP position as the end position for each candidate region. We repeated the sliding window analysis separately using *pcadapt* outliers identified in the full dataset and each geographic region‐specific dataset.

To compare patterns of population differentiation among candidate regions, we calculated pairwise *F*_ST_ using *pcadapt* outliers within each candidate region with *genet.dist* function (method=“WC84”) in *hierfstat* v.0.5.7 (Goudet, 2005). Significance was assessed by calculating 95% confidence interval of pairwise *F*_ST_ values using *boot.ppfst* function (nboot = 1000) in *hierfstat*. A pairwise *F*_ST_ value was considered significant if its confidence interval did not include zero. We further compared pairwise *F*_ST_ values calculated using *pcadapt* outliers in each candidate region and those with all SNPs and tested significance using Wilcox test. We also calculated population‐specific alternate/non‐reference allele frequencies (AF) of *pcadapt* outliers within candidate regions using PLINK v2.0 (Chang et al., 2015; Purcell et al., 2007). We visualized the difference in AF of *pcadapt* outliers using the R package *pheatmap* v1.0.12 (Kolde, 2019).

### 2.5 Linkage disequilibrium of candidate regions under selection

To estimate linkage disequilibrium (LD) of each candidate region, we calculated *r* ^2^ using PLINK v1.9 (‐‐ld‐window 999999999 ‐‐ld‐window‐kb 30000 ‐‐ld‐window‐r2 0.05) for SNPs with MAF ≥0.01 and located at least 400 bp from one another for each chromosome with a candidate region. We compared LD between *pcadapt* outliers within a candidate region and random SNPs (chromosomal background) on the same chromosome, visualized the differences using boxplots, and tested for significance using permutation tests with 10,000 iterations following the permutation test procedures described in Shi et al. (2021). Briefly, this involved drawing 10,000 samples of random SNPs equal to the number of *pcadapt* outliers within the candidate region to generate the null distribution for that chromosome. We then compared the empirical value using *pcadapt* outliers to the null distribution.

We visualized the overall LD pattern for each chromosome with a candidate region using the R package *LDheatmap* v0.99.7 (Shin et al., 2006). For each candidate region displaying strong LD (*r* ^2^ > 0.2) over an extended distance (>1 Mb), we conducted a series of post hoc analyses to examine whether it was a putative chromosomal inversion. First, we conducted a local PCA analysis using the R package *lostruct* (Li & Ralph, 2019) to examine the variation of population structure pattern across the corresponding chromosome. We divided the chromosome into non‐overlapping windows of 50 SNPs and used a 40‐dimension multidimensional scaling (MDS) analysis to examine whether the candidate region overlapped with windows with absolute loading values greater than three standard deviations above the average across all windows on the chromosome (Huang et al., 2020). Secondly, we conducted PCA using *pcadapt* outliers within the candidate region to identify whether individuals clustered in the distinct “three‐genotypic‐cluster” PCA pattern along PC1, a characteristic of chromosomal inversions (Lotterhos, 2019; McKinney et al., 2020). To assign individuals to a genotypic cluster, we applied k‐means clustering to the first two eigenvectors of the PCA using the function *kmeans* in the R package *adegenet* v2.1.3 (Jombart, 2008; Jombart & Ahmed, 2011). Lastly, to test for the development of divergent haplotypes due to reduced recombination in a chromosomal inversion, we examined haplotype structure within the candidate region by visualizing individual genotypes of *pcadapt* outliers using genotype heatmaps, where genotypes were color coded to represent homozygotes for alternate alleles (reference or alternative) or heterozygotes. We calculated the haplotype frequency of a chromosomal inversion in each population using the formula $F=\frac{2C_{0\;\text{or}\;2}+C_{1}}{2N}$ (Le Moan et al., 2021), where *C*_0_ or *C*_2_ is the number of individuals assigned to one of the homozygous clusters (cluster 0 or 2), *C*_1_ is the number of individuals assigned to the heterozygous cluster (cluster 1) in the PCA, and *N* is the number of individuals in each population. Without genome comparison with closely related species, it is difficult to conclude with confidence which haplotype represents the ancestral state or the derived state. We thus name them according to their haplotype frequency (major vs minor).

## 3 RESULTS

### 3.1 Rapture panel development

Preliminary RAD sequencing on 30 samples produced a total of 1,804,982,884 reads, of which 1,799,135,539 were retained. Following the STACKS pipeline and all quality control filters, a total of 150,000 loci were sent to Arbor Biosciences for bait development, of which baits were successfully designed for 105,073. We further removed loci with low/high GC content and retained a final panel of 100,000 loci with an average GC content of 44%.

### 3.2 Rapture sequencing

Demultiplexed rapture sequencing data of 951 baseline samples yielded a total of 8,211,065,462 retained reads, with an average of 8,634,138 retained reads per individual (range = 18,576–31,979,310 reads). Out of these reads, 86.7% (a total of 7,121,971,525 reads) were mapped to the European Alpine whitefish reference genome (GCA_902810595.1) in proper pairs with mapping quality greater than 20, with an average of 7,488,929 reads per individual (range = 12,739–28,082,161 reads). After quality filtering, the final dataset consisted of 829 baseline samples across 17 populations (Table 1) genotyped at the 197,588 SNPs across 92,725 loci. A summary of the number of SNPs and RAD loci retailed after each filtering step can be found in Table S3.

### 3.3 Population structure and genetic diversity

We identified multiple distinct genetic clusters even though the genetic differentiation across the full dataset was generally low (weighted *F*_*ST*_ = 0.0065). PCA showed the largest genetic break between the northwestern and eastern sides of the lake (Figure 1b) with the division located between Manistique (MNST) and Naubinway (NAUB). Therefore, we conducted additional PCA analyses on each lake region separately (Figure 1c–e). PCA on the northwestern side of the lake (Figure 1c) showed that populations in Big Bay de Noc (BBDN) and MNST as well as eastern Door County Peninsula, North/Moonlight Bays (NMNL) and Whitefish Bay (WTFB) clustered tightly together, along with some individuals sampled from southern Green Bay including Menominee River (MENO), Peshtigo River (PSTG), Larson's Reef (LRSF), Rileys Bay (RILY) and Fox River (FOXR). The rest of the individuals from southern Green Bay formed a separate large loosely clustered PCA group. Compared to the northwestern side, PCA on the eastern side (Figure 1d) showed greater genetic structure, with four genetic clusters identified. Ingalls Point (IGPT) and MSKG formed relatively distinct clusters. The third cluster contained individuals from Elk Rapids (EKRP) and Good Harbor (GOOD) and the fourth cluster contained individuals from the northeastern lake, including NAUB, Epoufette (EPFT), Cross Village (CXVL), and Little Traverse Bay (LTTV). An additional PCA on the northeastern Lake Michigan side identified two sub‐clusters (Figure 1e), with EPFT and NAUB being genetically similar, and CXVL and LTTV being genetically similar. ADMIXTURE results corroborated the patterns shown in PCAs, with support for K = 2 for all populations (Figure S1a) and populations from northwestern Lake Michigan (Figure S1b). In eastern Lake Michigan, K = 1 was most supported based on cross‐validation error (Figure S1c). However, examination of ancestry proportion from K = 1–10 showed that K = 5 seemed to make the most biological sense (Figures S1c and S2). Pairwise *F*_*ST*_ values between northwestern populations were low, with an average of 0.001, ranging from 0 to 0.005, with many pairwise values being statistically indistinguishable from 0. In comparison, pairwise *F*_*ST*_ values between eastern populations were higher, with an average of 0.006, ranging from 0 to 0.013 (Figures S3).

**FIGURE 2:**
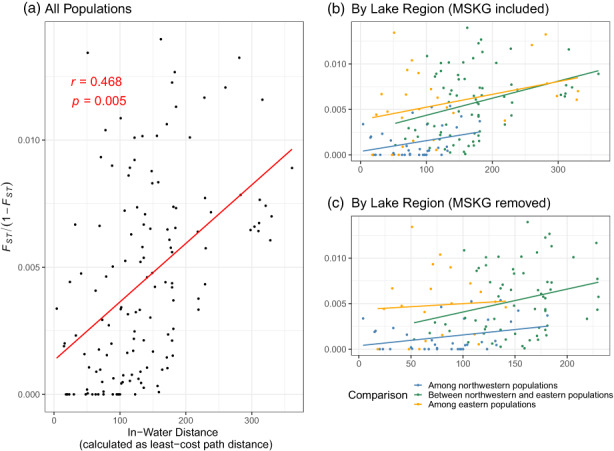
Correlation of linearized pairwise *F*_ST_, *F*_ST_/(1−*F*_ST_), with the pairwise in‐water distance (unit not specified) among all population pairs (a), populations within the same side of the lake (b), and population within the same side of the lake but with MSKG removed (c). Isolation by distance (IBD) was assessed using mantel tests with 10,000 permutations.

There was a significant signal of IBD when all populations were considered (Figure 2a; Mantel test *r* = 0.468, *p* = 0.005) and the IBD signal remained significant after MSKG was removed (Mantel test *r* = 0.375, *p* = 0.0003). However, when considering populations in each lake region separately, the correlation between genetic distance and in‐water distance became nonsignificant (northwestern region: mantel test *r* = 0.300, *p* = 0.097; eastern region: mantel test *r* = 0.359, *p* = 0.204). Overall, similar to the pattern from Figure S3, the pairwise differentiation among eastern populations was higher than those in the northwestern lake regardless of whether MSKG was included or not (Figure 2b,c). Genetic diversity estimates were similar across populations (Table 1) with an average *H*_E_ of 0.124 (0.117 to 0.134), *H*_O_ of 0.154 (0.144 to 0.177), and *F*_IS_ of −0.064 (−0.101 to −0.046). Estimates of *N*_e_ for all populations were infinity, highlighting the challenges of obtaining non‐infinite *N*_e_ estimates in large populations(Waples & Do, 2010).

### 3.4 Identification of Genomic Islands of adaptive divergence

**FIGURE 3:**
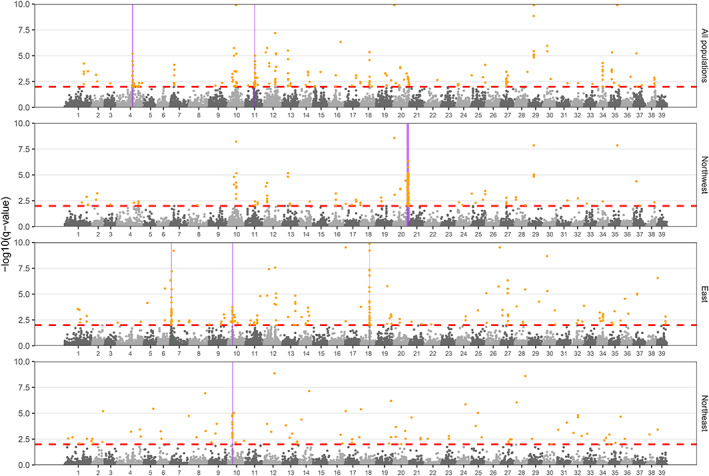
Genome scan analyses using *pcadapt* on the whole dataset and each geographic region‐specific dataset (northwestern, eastern, and northeastern Lake Michigan). Orange points are *pcadapt* outliers with adjusted *p* values (*q* values) less than 0.01 (red dashed line). Six candidate regions under selection are highlighted in purple (see Table S4 for details). The y‐axis was restricted to the range 0–10 for visualization purpose.

A total of 566 outlier loci were identified by *pcadapt* (“*pcadapt* outliers”) in the full dataset and each region‐specific dataset combined (Table S2). These *pcadapt* outliers were not evenly distributed across the genome (Figure 3). Using sliding window analyses conducted separately with *pcadapt* outliers in the full dataset and each geographic region‐specific dataset, we identified six candidate genomic regions of adaptive divergence on six chromosomes (chromosomes 4, 7, 10, 11, 18, and 20; Table S4; Figure 3). Most regions were only identified in a single dataset, but the region on chromosome 10 was identified in both the eastern and northeastern datasets (Figure 3). These six genomic regions were relatively large, ranging from 1.2–8.5 Mb, and included 12–85 *pcadapt* outliers each (Table S4).

**FIGURE 4:**
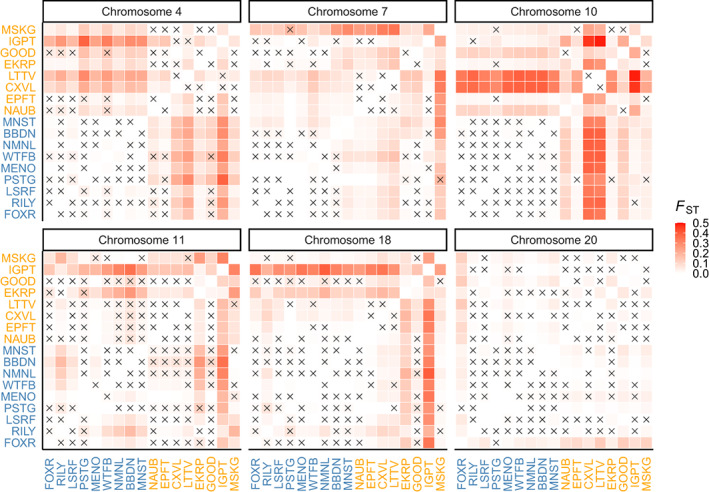
Pairwise *F*_ST_ heatmap between 17 lake whitefish populations in Lake Michigan using *pcadapt* outliers in each candidate region shown in Figure 3. Non‐significant pairwise *F*_ST_ values were labeled with an “X”. the number of *pcadapt* outliers in each candidate region is 12 (chromosome 4), 23 (chromosome 7), 24 (chromosome 10), 16 (chromosome 11), 22 (chromosome 18), and 85 (chromosome 20), respectively.

Comparisons of pairwise *F*_ST_ heatmaps using *pcadapt* outliers among these six candidate regions showed a mosaic pattern of population differentiation, with different genomic regions distinguishing different populations (Figure 4). Specifically, northwestern populations were generally differentiated from eastern populations in the candidate region on chromosome 4. MSKG was highly differentiated from other populations in the candidate region on chromosome 7, whereas IGPT was distinct in the region on chromosomes 11 and 18. CXVL and LTTV were genetically similar but highly different from other populations in the region on chromosome 10. The pattern in the region on chromosome 20 was less clear and pairwise *F*_ST_ values were lower for this region compared to the others. Overall, pairwise *F*_ST_ values among population pairs using *pcadapt* outliers within each candidate region were significantly higher (*p* < 0.01, Wilcox test) than those using all SNPs in the full dataset except for the region on chromosome 20 (Figure S4).

Comparisons of allele frequencies (AF) of *pcadapt* outliers among these six candidate regions (Figure S5) corroborated the patterns described above and further demonstrated the mosaic of population differentiation across candidate regions. In the region on chromosome 4, mean AF of *pcadapt* outliers in northwestern populations was 0.51, whereas that in the eastern population was 0.39. In the region on chromosome 7, mean AF of *pcadapt* outliers in MSKG was 0.47, whereas that in other populations were 0.23. Similarly, *pcadapt* outliers in the regions on chromosomes 11 & 18 had high AF in IGPT (0.41 and 0.44, respectively), but low AF in others (0.14 and 0.10, respectively). In the region on chromosome 10, the mean AF of *pcadapt* outliers was high in CXVL (0.47) and LTTV (0.47), but low in others (0.22). The pattern in the region on chromosome 20 was less clear, with only subtle allele frequency differences among populations.

### 3.5 Linkage disequilibrium of candidate regions under selection

Across all six candidate regions, mean values of *r* ^2^ between *pcadapt* outliers were generally higher compared to the chromosomal backgrounds (Figure S6), though the differences were not significant except for the region on chromosome 20 (see Table S4 for detailed permutation test results). This region had elevated LD (mean *r* ^2^ = 0.32) extending almost its entire ~8.5 Mb region from 45.4 to 53.8 Mb (Figure S7). By contrast, no large LD blocks were observed in other candidate regions (Figure S7).

**FIGURE 5:**
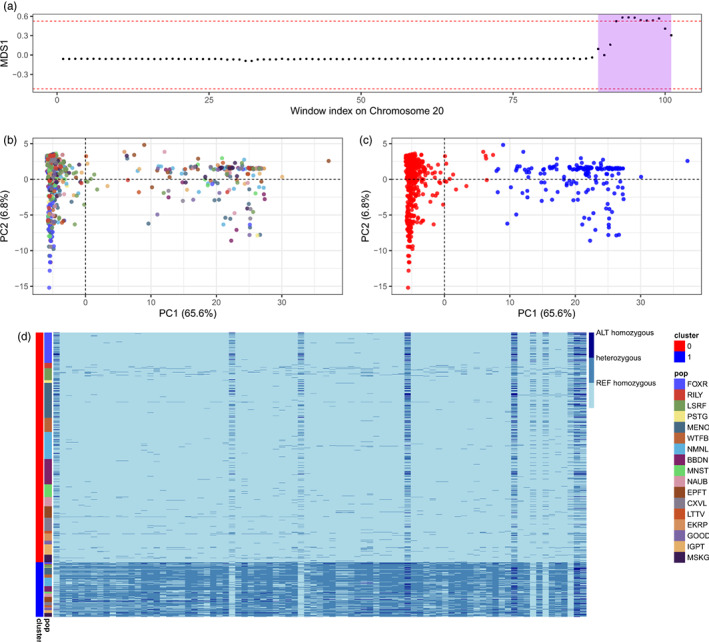
Putative chromosomal inversion on chromosome 20 (45.4–53.8 Mb). (a) *Lostruct* identified a series of eight windows with extremely high loading values along MDS1 within this region (purple shade); (b) and (c) PCA using 85 *pcadapt* outliers within this region showed that individuals were grouped into two clusters using k‐means clustering (cluster 0 and 1) along PC1 and the grouping pattern was not associated with populations. (d) Genotype heatmap using 85 *pcadapt* outliers showed clear haplotype structure within this region. Rows represent individuals, which are color ordered by PCA clusters (cluster 0 and 1); columns represent SNPs, which are ordered by chromosomal positions.

Within the candidate region on the chromosome 20, *lostruct* identified a series of eight windows with extremely high loading values along MDS1 (Figure 5a), suggesting there was a significant difference in population structure patterns between this region and the rest of chromosome 20. Using 85 *pcadapt* outliers within the candidate region on chromosome 20, PCA showed that individuals were grouped into two clusters (cluster 0 and 1) along PC1 and the grouping pattern was not associated with populations (Figure 5b,c). This candidate region also showed a clear haplotype structure (Figure 5d), with 670 individuals being homozygous for the reference allele (cluster 0) and 159 individuals being heterozygous (cluster 1). The above evidence suggests that this region may be a putative chromosomal inversion. The minor haplotype of this putative inversion was very rare with an estimated overall haplotype frequency of 9.6% and similar frequencies across populations, ranging from 2.7 to 21.1%. No homozygotes were observed for the minor haplotype.

## 4 DISCUSSION

### 4.1 High‐density genomic data reveals novel insights of population structure

Our study represents the most extensive population genetic study on lake whitefish in Lake Michigan to date. Though patterns of genetic structure were generally similar between our study and VanDeHey et al. (2009), our assessment provided several new and important insights: (1) a large genetic break between the northwestern and eastern sides of Lake Michigan; (2) a much greater level of population structure on the eastern side compared to the northwestern side; (3) less differentiation between NMNL and BBDN than reported by VanDeHey et al. (2009); and (4) two putative genetic clusters in northwestern Lake Michigan that appear to be mixed in central Green Bay.

The largest genetic break we observed was between the northwestern and eastern sides of Lake Michigan, with a division between MNST and NAUB, indicating limited gene flow between the two sides of the lake. Results from our study indicate that northwestern and eastern populations are differentiated by both neutral and putatively adaptive markers, as populations on each side of the observed genetic break display relatively high overall *F*_ST_ values and are also differentiated at the genomic island of divergence identified on chromosome 4. Using three polymorphic isozymes, Imhof et al. (1980) reached a similar conclusion that lake whitefish east of Seul Choix Point (in between MNST and NAUB) did not interbreed freely with those to the west. Tagging data also indicated limited movement between the two sides of Seul Choix Point (Ebener et al., 2010). Despite these observations, we are unsure which environmental factors could explain the limited movement between the two sides of the lake. Future studies are warranted to explore candidate environmental variables to fill this gap via a comprehensive genotype‐environment association analysis, which is beyond the scope of the current study.

In addition, we demonstrated a much greater level of population structure on the eastern side of the lake compared to the northwestern side, potentially reflecting greater levels of habitat heterogeneity on the eastern side. There is much more variation in water depth on the eastern side compared to the northwestern side according to Lake Michigan Bathymetry data (https://www.ngdc.noaa.gov/mgg/greatlakes/lakemich_cdrom/html/geomorph.htm), as well as more variation on the northeastern side compared to the southeastern side. Other factors may play a role, such as shoreline structure, bottom type, and riverine inputs (Janssen et al., 2005; Turschak et al., 2019), as the nearshore environment of Lake Michigan is highly variable spatially (Vadeboncoeur et al., 2011).

Within northwestern Lake Michigan, we found NMNL and BBDN were genetically similar. Though VanDeHey et al. (2009) detected a significant pairwise *F*_*ST*_ value between NMNL and BBDN, it was very low (*F*_*ST*_ = 0.0025), and these two populations were the last to be divided into separate genetic groups, suggesting difficulty in discriminating between them. In addition, the genetic differentiation between NMNL and BBDN does not seem to be temporally stable (Nathan et al., 2016), which further supports substantial gene flow between these populations. Our results also indicate that there are likely two weakly differentiated genetic clusters within northwestern Lake Michigan: BBDN/MNST/eastern Door County Peninsula (NMNL & WTFB) and southern Green Bay (MENO, PSTG, LRSF, RILY and FOXR). This pattern could be due to the recent recolonization of the river habitats in southern Green Bay by a remnant population of southern Green Bay reef spawners. These two clusters might also be from different glacial lineages that were separated during the Wisconsin glaciation and began to make secondary contact during postglacial colonization. Although the general formation and geomorphic evolution of the Great Lakes basin following deglaciation is relatively well understood (April et al., 2012; Homola et al., 2021), little is known about the fish colonization history in a specific Great Lake such as Lake Michigan. Future studies could further investigate the metapopulation dynamics of lake whitefish in Green Bay using a combination of otolith microchemistry (Doerr et al., 2021), tagging, genomics and demographic modeling (Rougemont et al., 2020).

### 4.2 Pronounced Genomic Islands of adaptive divergence in a high gene flow species inhabiting heterogeneous environments

We observed contrasting signals of population structure between all SNPs in the full dataset and *pcadapt* outliers within each island of divergence, with generally much higher population differentiation using *pcadapt* outliers within each island of divergence compared to all SNPs. While the chromosome 4 island displayed relatively similar patterns of population structure as the full dataset, the other islands did not, and instead largely differentiated single populations or population pairs from others. For example, the chromosome 7 island strongly differentiated MUSK, the most southern population in the study, from all others. Muskegon displayed moderately high differentiation in the full dataset but was by no means the most differentiated population in the study. The islands on chromosomes 10, 11, and 18 differentiated single or pairs of eastern Lake Michigan populations from most others in the study. While these eastern populations did display relatively high differentiation in the full dataset, the pattern of extremely high differentiation at a single population or a population pair was not present in the full dataset. We hypothesize that these patterns of extreme differentiation at islands of divergence present in a small number of populations reflect fine‐scale local adaptation potentially facilitated by these islands.

Both theoretical simulation studies and empirical studies have shown that increased gene flow will lead to the increasingly concentrated genomic architecture of local adaptation (Shi et al., 2021; Via, 2012; Yeaman, 2013; Yeaman & Whitlock, 2011). In a species with high gene flow across heterogeneous environments, adaptive differentiation is often restricted to genomic islands of divergence that can resist the homogenizing effects of gene flow (Via, 2012). Our data suggest that gene flow in lake whitefish is high, given the low overall *F*_*ST*_ (weighted *F*_*ST*_ = 0.0065) and low pairwise *F*_*ST*_ values (*F*_*ST*_ = 0–0.0138). Systematic estimation of lake whitefish spawning site fidelity at a broad scale is lacking, though tagging data suggest some lake whitefish exhibit spawning site fidelity (Ebener et al., 2010). However, this fidelity is much lower than other species such as Pacific salmon (*Oncorhynchus* spp.), and straying to neighboring spawning populations (Ebener et al., 2021) can result in high gene flow. Despite gene flow, the pronounced genomic islands that we observed suggest that heterogeneity of spawning habitats has led to adaptive divergence, even across small spatial scales (10s of km) on the eastern side of the lake. This pattern of multiple islands of divergence facilitating adaptation to a variety of heterogenous habitats is similar to what has been observed in species such as prairie sunflower (*Helianthus petiolaris*), where several genomic islands of divergence were found throughout the genome (Huang et al., 2020; Todesco et al., 2020).

Besides the divergence hitchhiking theory as described above (Via, 2012), heterogeneous genome‐wide divergence can also be caused by linked selection regardless of gene flow, resulting in a reduction in nucleotide diversity in the vicinity of the sites targeted by selection (Burri et al., 2015; Cruickshank & Hahn, 2014). In addition, genomic islands can be caused by global adaptation in structured populations, where the spread of a uniformly beneficial mutation leaves a spatially variable pattern in the allele frequency across the range of the species as a consequence of incomplete sweeps or recombination during a sweep (Booker et al., 2021). These alternative hypotheses may be distinguished by examining patterns of nucleotide diversity within (*π*) and between populations (*d*_XY_), which can be accurately calculated using a sliding window‐based approach with all sites included (variant and invariant sites) such as the approach implemented in *pixy* (Korunes & Samuk, 2021). We did not attempt to disentangle these competing theories due to the characteristics of Rapture data (i.e., a low percentage of the genome is sampled compared to whole genome sequencing and extracting invariant sites is difficult). In addition, a recent simulation study has demonstrated that there is no universally diagnostic signature of local adaptation based on nucleotide diversity within populations, which can be decreased or increased depending on the relative strengths of migration and selection (Jasper & Yeaman, 2020), further complicating this debate. Other factors such as variation in recombination, mutation rate, and gene density can also lead to a heterogeneous genomic landscape of differentiation (Ravinet et al., 2017; Schumer et al., 2018), but exploring these is beyond the scope of the current study.

The six candidate regions that we identified showed elevated LD compared to the chromosomal background. Particularly, the region on the chromosome 20 (45.4–53.8 Mb) was a large LD block, which showed a distinct population structure pattern compared to the rest of the chromosome and a clear haplotype structure, suggesting a putative chromosomal inversion. The minor haplotype of this putative inversion was very rare (overall haplotype frequency of 9.6%), with similar frequencies across populations (2.7%–21.1%). Unlike a typical chromosome inversion with three genotypic clusters (Lotterhos, 2019; McKinney et al., 2020), we did not observe any homozygotes for the minor haplotype. This is probably due to a combination of factors, e.g. low frequency of minor haplotype and the number of samples we sequenced. Additional samples might reveal that they are present but in extremely low frequency. Interestingly, an independent genomics study of lake whitefish identified the same putative inversion on chromosome 20 and documented evidence that it had three genotypic clusters along PC1 and was highly differentiated among spawning populations in Lake Erie (Euclide et al., 2022). It is important to note that our results only suggest a putative inversion. Future studies could take advantage of the recently released long‐read genome assemblies of lake whitefish (Mérot et al., 2022) to verify the existence of this putative chromosomal inversion.

### 4.3 Management implications and conclusions

The pronounced genomic islands of adaptive divergence that we observed highlight the importance of considering adaptive variations when examining the genome‐wide genetic variation, particularly for managed species with high gene flow across heterogeneous environments. In the case of lake whitefish in Lake Michigan, different genomic islands of adaptive divergence distinguished different populations, suggesting that combinations of genotypes at these adaptive regions are facilitating local adaptation to a variety of heterogenous habitats, especially in eastern Lake Michigan. These data provide evidence that there are meaningful functional differences among the populations on the east side of the lake that may warrant distinct management strategies and a more conservative approach to preserve the basin‐wide stability and evolutionary potential of the species. Our results suggest that gene flow among lake whitefish populations is common across much of Lake Michigan, but divergence at putatively adaptive loci observed between even geographically proximate populations (separated by 10s of km) indicates local adaptation plays a central role in maintaining high frequencies of particular adaptive genetic variants in specific populations. Management agencies could consider actions that extend location‐specific harvest closures to ensure certain stocks are protected during spawning, despite interannual variability in the timing of spawning. However, given that most lake whitefish harvest occurs outside of spawning, future mixed stock analyses could be used to establish the spatial range of each stock throughout the year to enable stock‐based management in favor of current geographically delineated management boundaries. The outlier SNPs uncovered here provide ample power for future SNP panel design that would permit such research to be efficiently performed.

Anthropogenic selection pressure such as fishing can lead to genetic changes on top of natural selection. Though fisheries‐induced evolution might be a slow and less obvious process on decadal time scales, it is often unavoidable even with optimal management practices (Eikeset et al., 2013; Law, 2007). Therefore, fishery management may benefit from increased consideration of the evolutionary consequences of harvest (Jørgensen et al., 2007). Genetic monitoring provides a promising tool to quantify temporal genetic changes and detect genetic erosion (Hoban et al., 2021; Schwartz et al., 2007). Our comprehensive genomic assessment of neutral and adaptive genetic variations of lake whitefish populations in Lake Michigan can serve as a baseline genomic resource for future research. The putatively adaptive loci we identified hold great promise to significantly improve the power to detect changes in functional genetic variation that contributes to the spatially complex local adaptation patterns of lake whitefish. Collectively, our work provides a baseline understanding of the neutral and adaptive genetic structure of lake whitefish populations in Lake Michigan, uncovers complex spatial and genomic patterns, and sets the stage for better integration of genetic diversity in management efforts for this culturally, economically and ecologically important native species in the Great Lakes.

## CONFLICT OF INTEREST

The authors declare no conflict of interest.

## Supporting information


Figure S1

Figure S2

Figure S3

Figure S4

Figure S5

Figure S6

Figure S7

Tables S1–S5

## Data Availability

Demultiplexed initial RAD sequencing data (*N* = 30) and Rapture data (*N*  951) used in this study are archived in the NCBI Sequence Read Archive with a BioProject ID, PRJNA687593. The Rapture data also included additional 175 individuals with mixed stock origins for a different study, and these 175 samples were sequenced and processed together with 951 baseline samples. Sample meta information along with sequence accession numbers for all samples can be found in Table S5. A *fasta* file for the Rapture panel with 100 K baited loci and a *vcf* file after filtering (829 individuals and 197,588 SNPs) are archived on https://doi.org/10.5061/dryad.r4xgxd2gq (line TBD). Other intermediate data files and all bioinformatic scripts supporting this article are available on the Github repository (https://github.com/melodysyue/lwf_LM_popgen). Questions pertaining to data generated for this project should be directed toward the corresponding author.
