# Supplementary figures and images for: High‐density genomic data reveal fine‐scale population structure and pronounced islands of adaptive divergence in lake whitefish (Coregonus clupeaformis) from Lake Michigan
Source: Evol Appl. 2022 Sep 20;15(11):1776–91. doi: 10.1111/eva.13475 (PMC9679245; doi:10.1111/eva.13475)

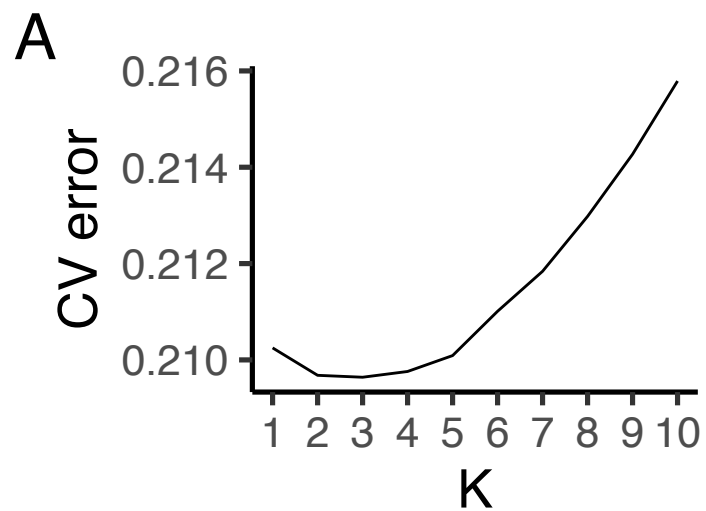

All Populations (K=2)

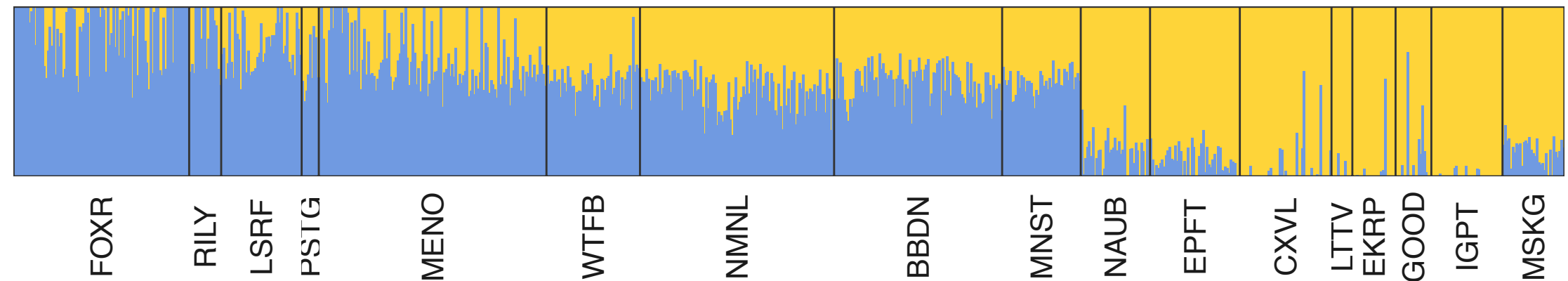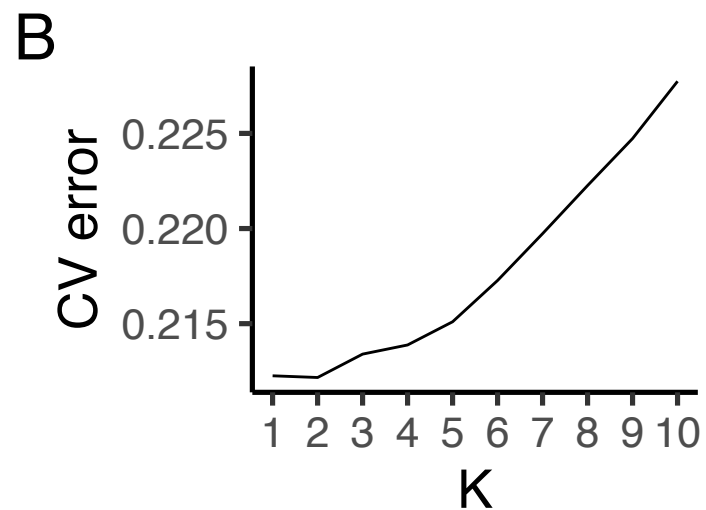

Northwestern Lake Michigan (K=2)

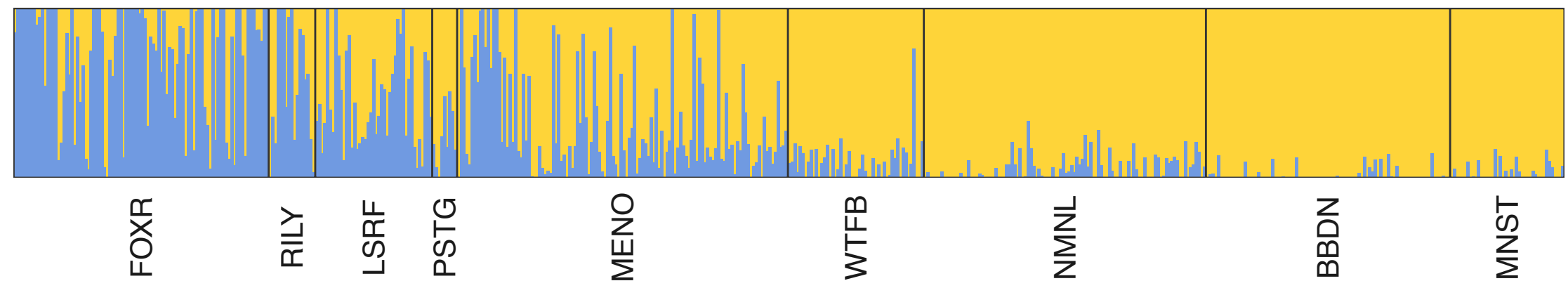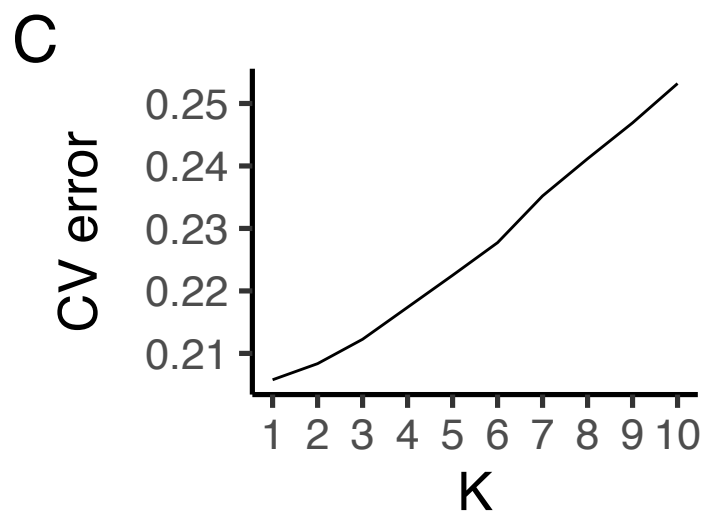

Eastern Lake Michigan (K=5)

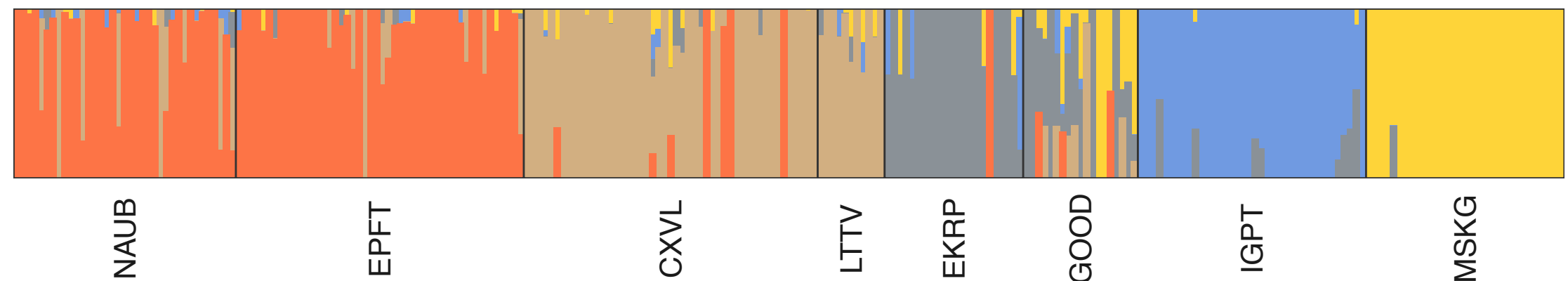

Supplement: Supplementary file 1 — Figure S1 [file EVA-15-1776-s004.pdf]

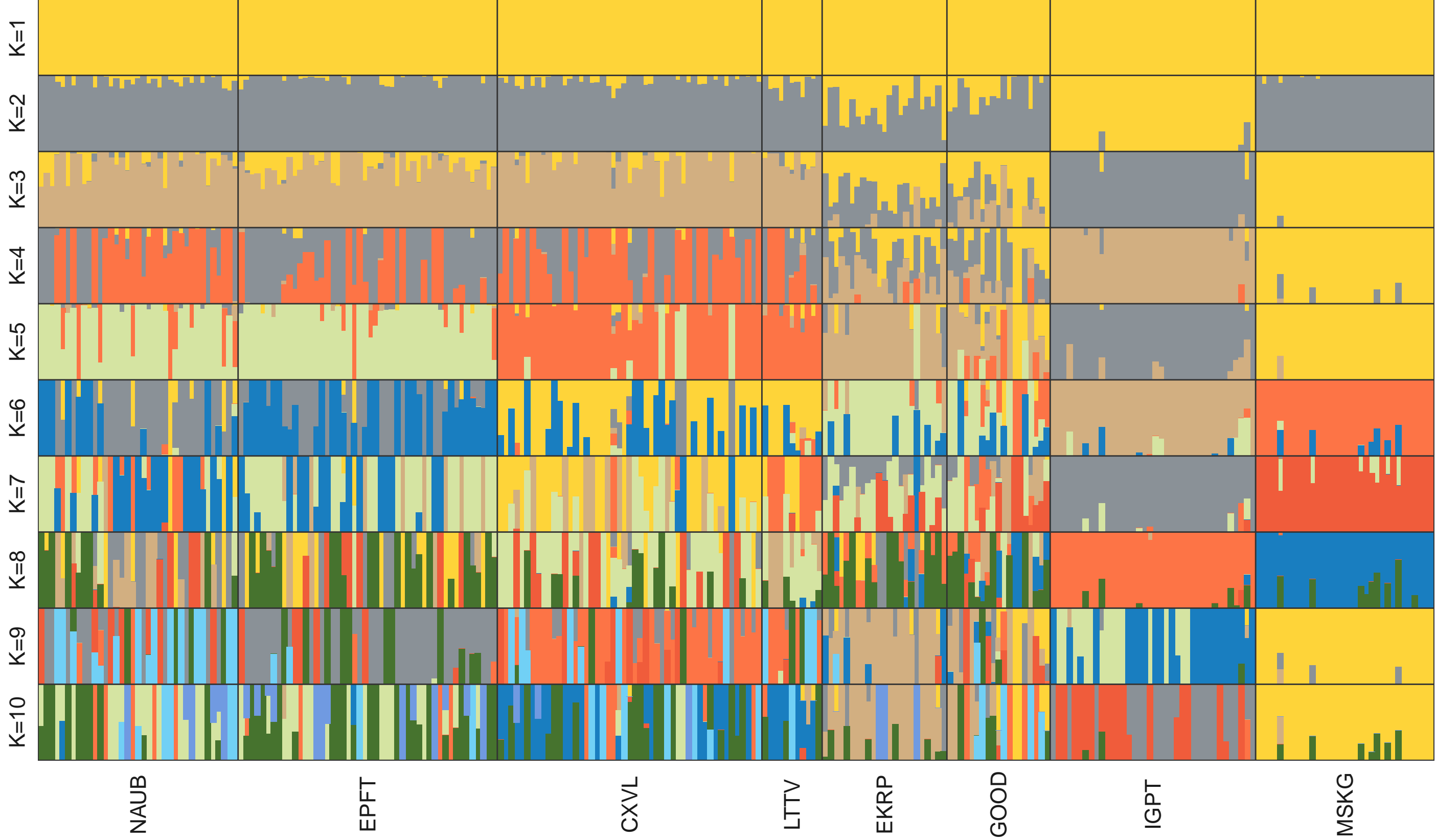

Supplement: Supplementary file 2 — Figure S2 [file EVA-15-1776-s008.pdf]

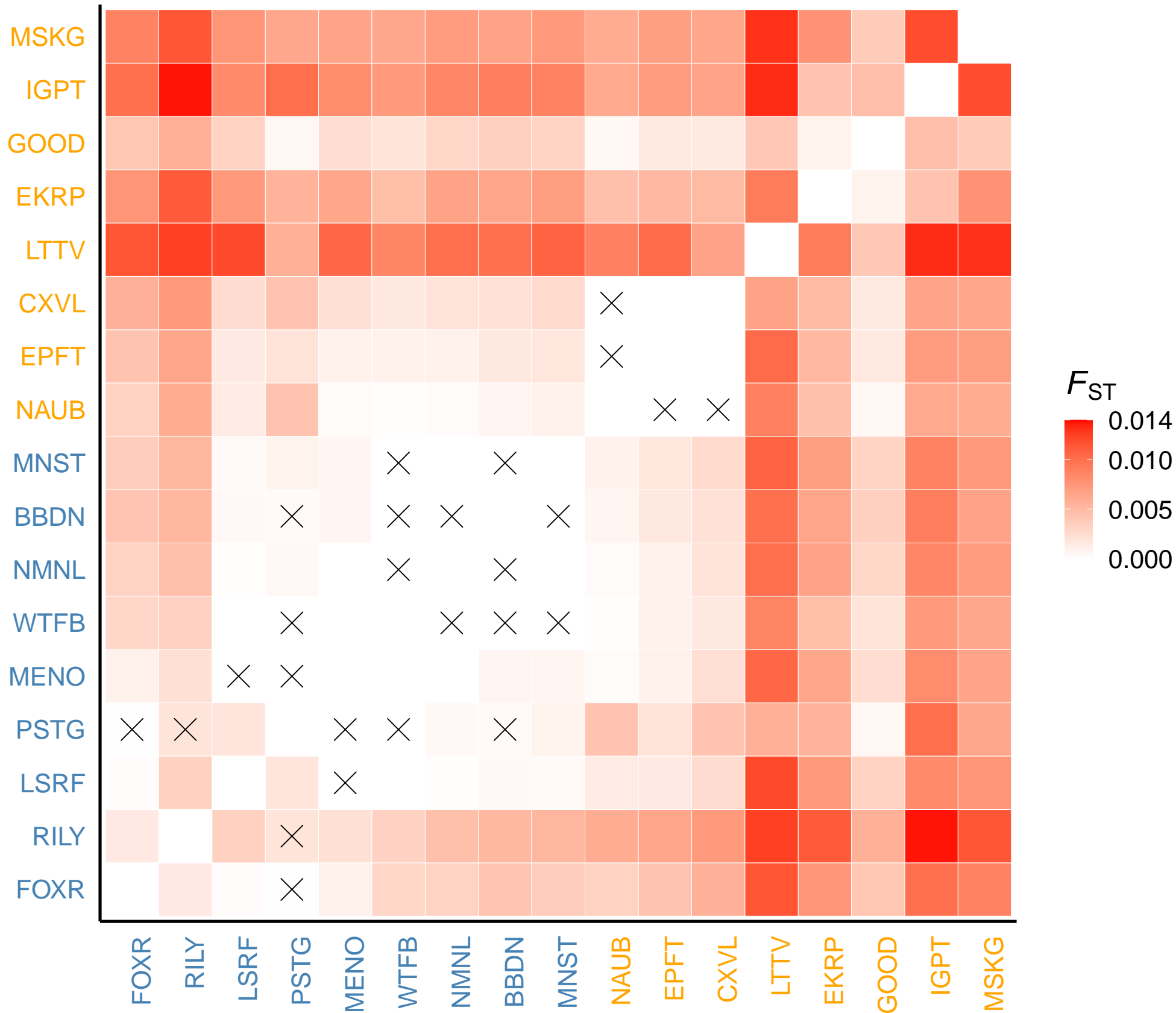

Supplement: Supplementary file 3 — Figure S3 [file EVA-15-1776-s007.pdf]

Pairwise  $F_{ST}$

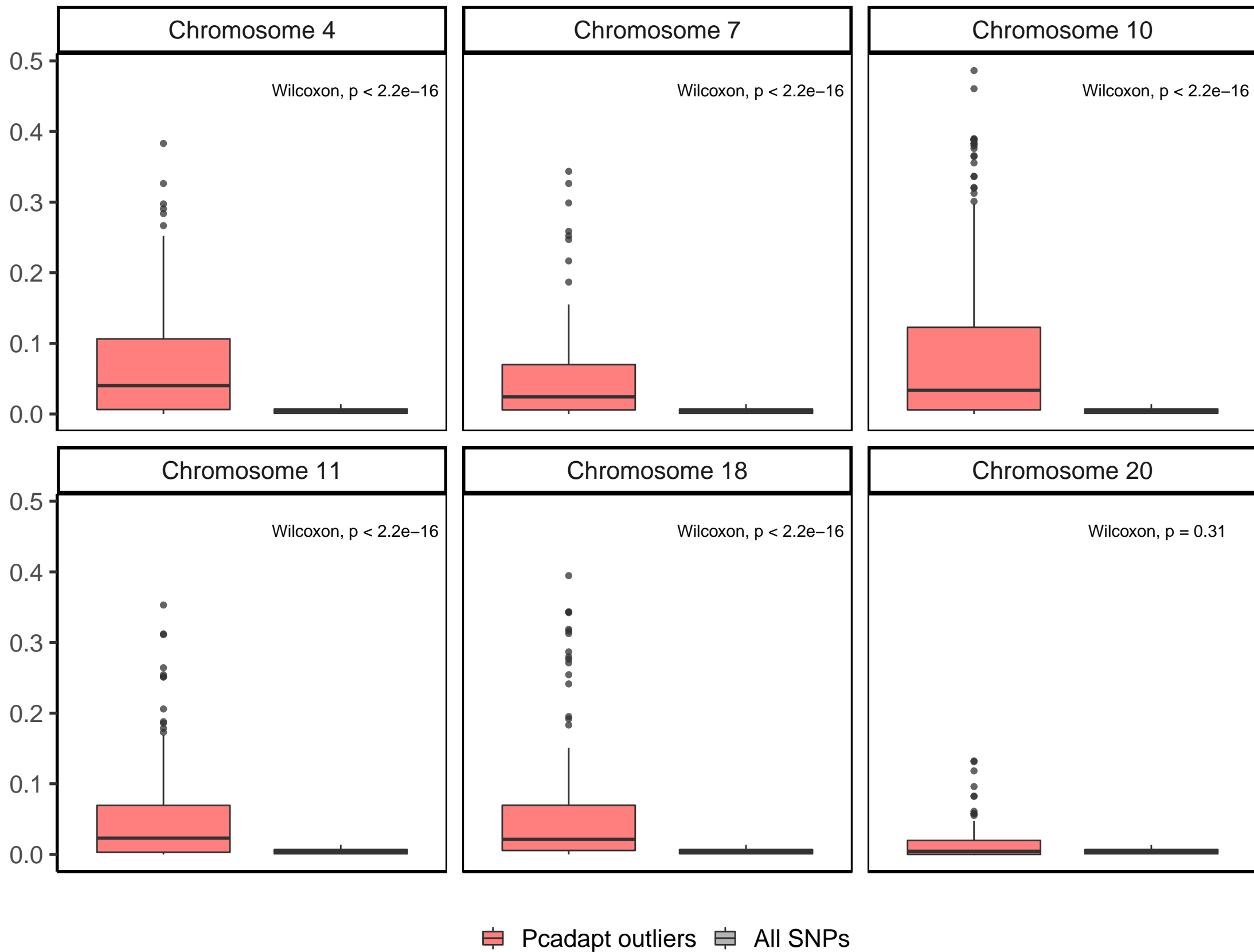

Supplement: Supplementary file 4 — Figure S4 [file EVA-15-1776-s003.pdf]

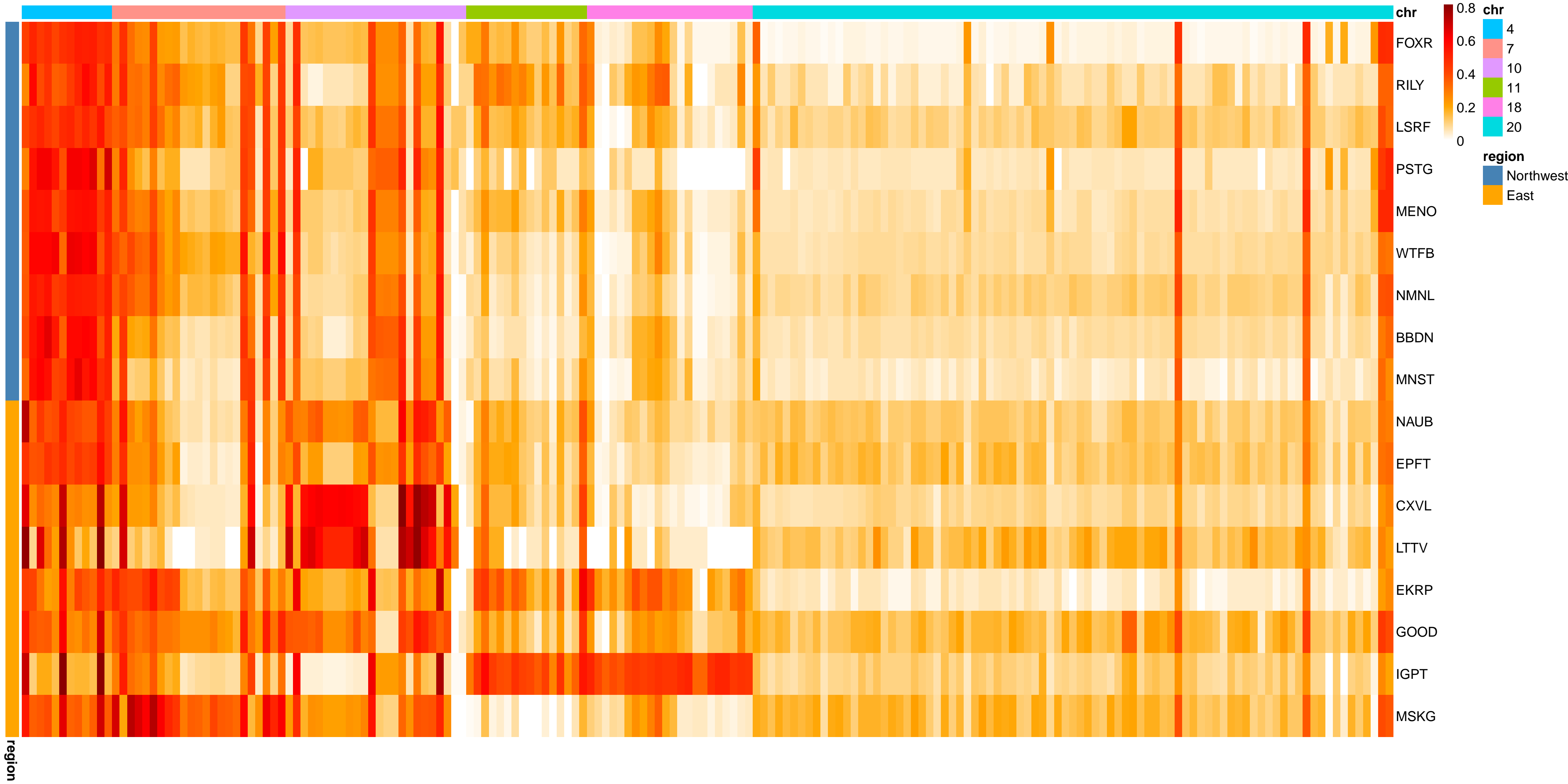

Supplement: Supplementary file 5 — Figure S5 [file EVA-15-1776-s001.pdf]

$r^2$

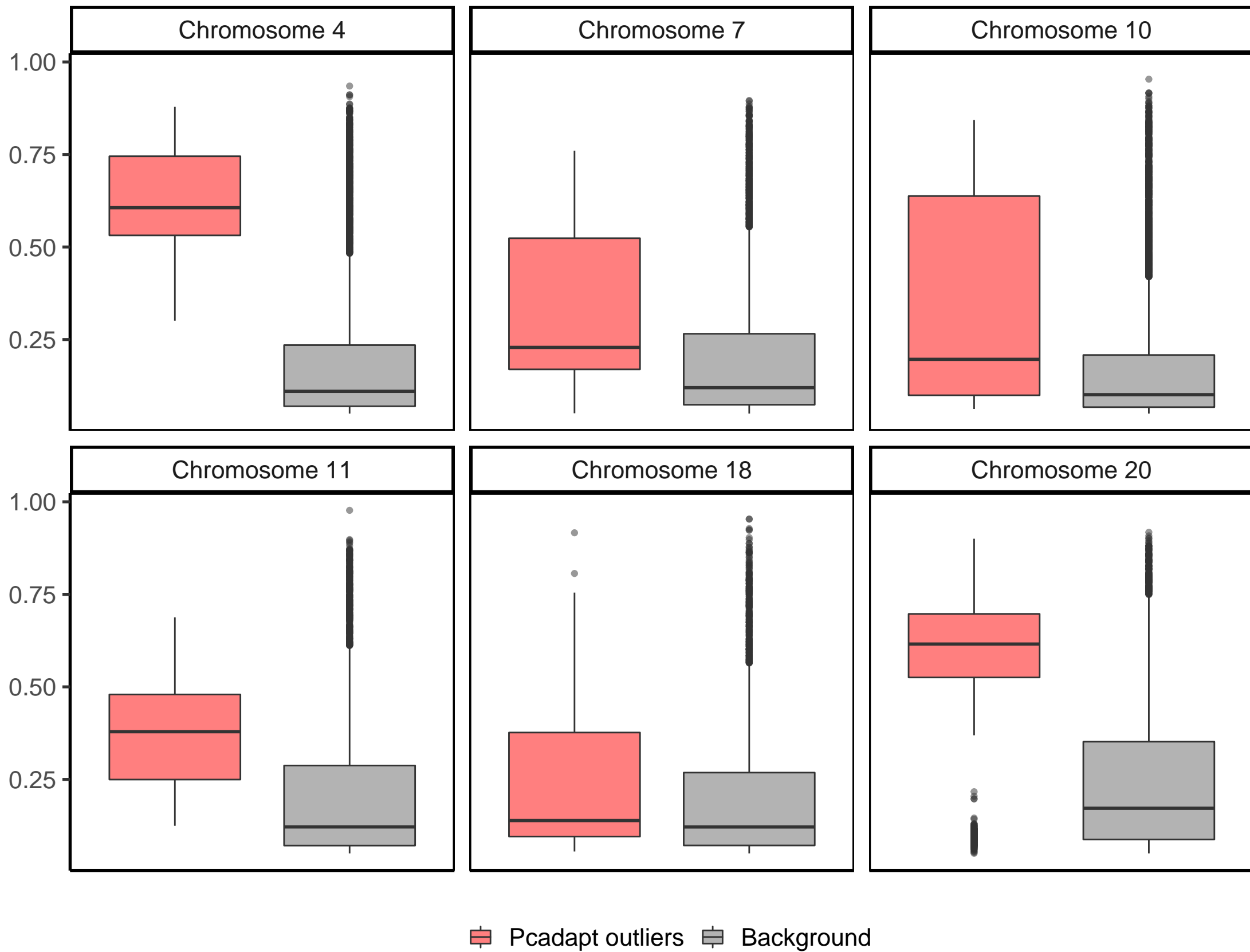

Supplement: Supplementary file 6 — Figure S6 [file EVA-15-1776-s002.pdf]

Chromosome 4

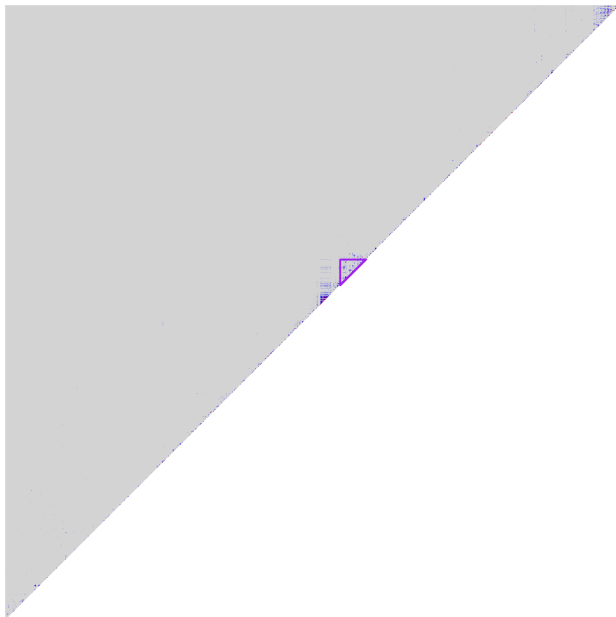

Chromosome 7

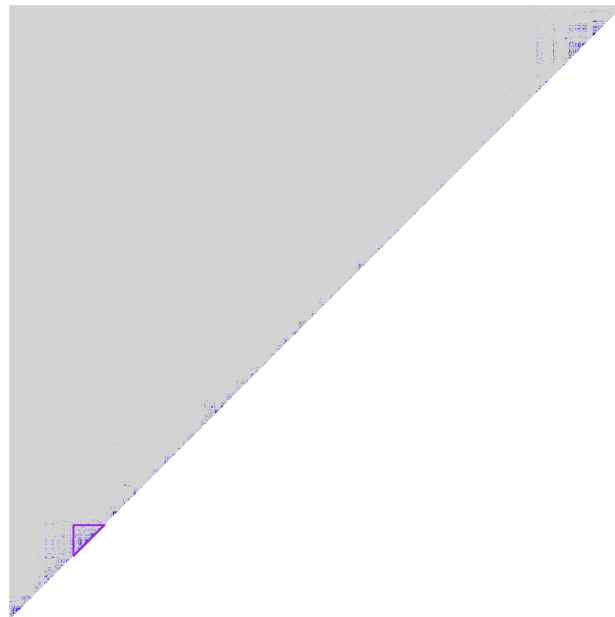

Chromosome 10

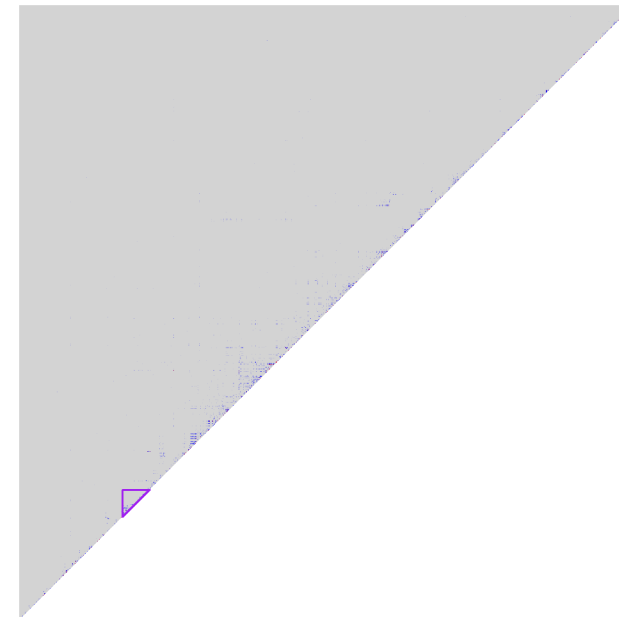

Chromosome 11

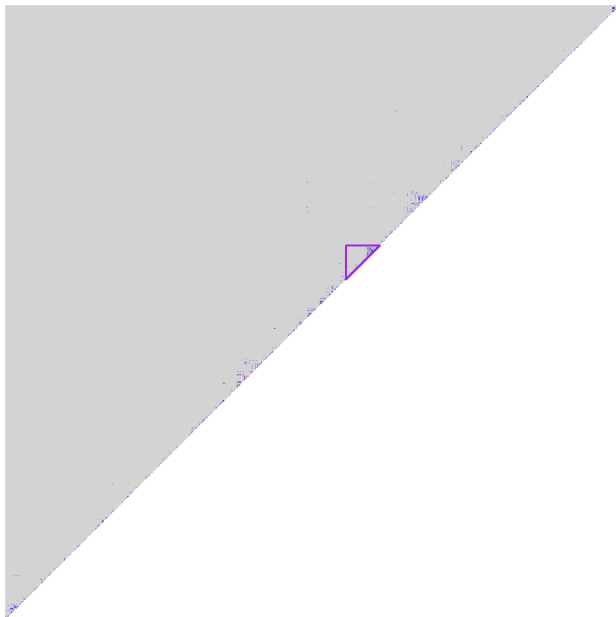

Chromosome 18

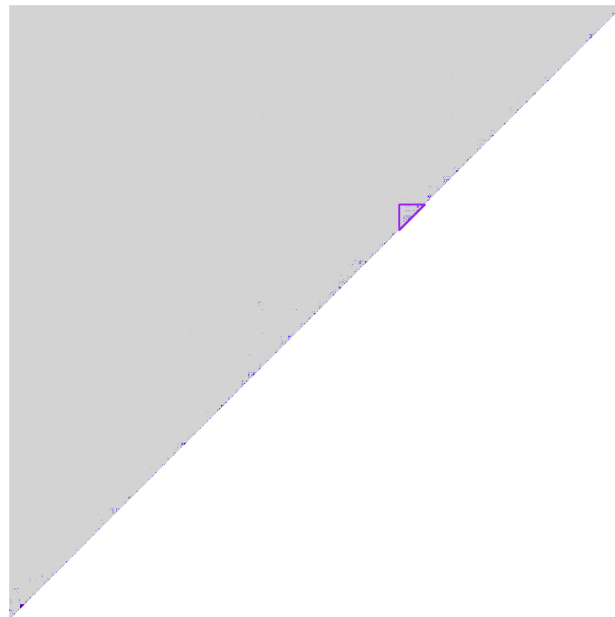

Chromosome 20

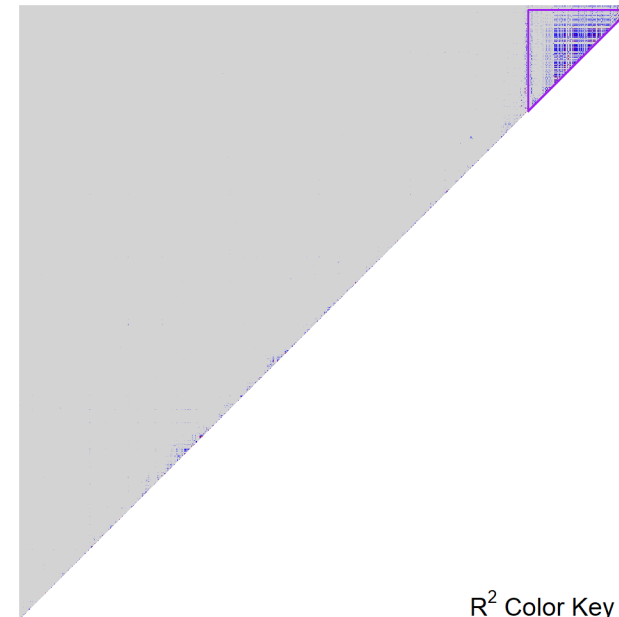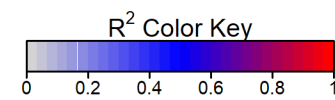

Supplement: Supplementary file 7 — Figure S7 [file EVA-15-1776-s005.pdf]
